# Singular Freak in Nature, or Malformation

**Published:** 1864-01

**Authors:** D. O. Crist

**Affiliations:** Bloomington, Ill.


ARTICLE II.

### SINGULAR FREAK IN NATURE, OR MALFORMATION.

BY D. O. CRIST, M.D., of Bloomington, Ill.

On the morning of the 27th of January, 1858, I was called to see Mrs. D., a lady of much respectability, in her first confinement. On entering the lady's chamber, seeing her mother leaving the bed, and Mrs. D. apparantly easy, I supposed at once that I had arrived too late, as the old ladies say. But a few moments stay convinced me that my conjecture was wrong. Although there was, as is generally the case in first confinements, a cessation of the pains. But, however, in a proper time they returned; and I found them to be of that character so minutely described by CHURCHILL as the first stage of labor. Although her husband told me when he came that she had been suffering for fifteen hours, and was very bad. I found the pains quite regular, and to all appearance efficient. After proper time and observation, I proposed an examination per vaginum, which was acceeded to, though not without some fear on the part of the lady, as is generally the case in first confinements; but, however, by the assurance of the mother, they were soon removed, and I proceeded with the examination, and found that labor had commenced, and the parts were well lubricated and of proper temperature, and the dilatation of os uteri was good; and finding quite a large and roomy pelvis, I anticipated a speedy delivery, but found, on extending the examination, it proved to be a footling presentation. The membranes were not ruptured, nor did they protrude as they generally do in head or natural presentations. Being always cautious, in cases of that character, not to rupture the bag of waters, for fear that the feet may pass down, and the labor become more difficult by a prolapse of the cord. However, finding the labor not as far advanced as I expected previous to the examination, I excused myself for one hour; and not being able to get back by the time appointed, they had dispatched a messenger for me.

When I returned, I found the pains more efficient, and the membranes ruptured, and the child entering inferior strait; and in two hours' time she was delivered of a healthy but rather delicate female child. Finding the circulation in the funis active, I separated the child after ligating the cord twice, thinking from the appearance of abdomen and smallness of the child there must certainly be a second child. After the proper attention to the babe, I found, by taking hold of the cord, that it was scarcely long enough to extend externally to the vulva. But by making an examination, I found what I supposed to be another child, and it proving to be a footling also. After acquainting the grandmother (for such she was,) of the fact, of their being another child as I supposed, I then acquainted the lady herself of the probability of her being a mother again soon, as there appeared to be another within the uterus; assuring her that all that was necessary for her welfare was for her to keep quiet, and I thought she would soon be delivered of a second child. However, the pains not coming on for some time, I ordered a decoction of secale cornuetum; thinking, in case of hemorrhage, that I would be prepared to meet the urgency of the case.

In the course of an hour from the time the first child was born the pains returned quite active, and in a few moments she was delivered of a second child, with the following singular and remarkable appearance:—

First in order comes the head, which was quite small in comparison to the body, not having any bones within, but covered tolerably well with hair, without the appearance of any eyes, but a small aperture for the mouth, or seemingly where it should be, this aperture extending down into the thoracic region. Not having opened the thorax, I cannot say if the lungs were well developed or not.

The neck was short, and almost as large in circumference as the head. The thorax was well developed in every respect, with the exception of arms, and there was not the least sign of arms, nor was there anything to correspond. The breasts or nipples were fully formed and quite prominent, much more so than natural.

The cord was rather singular, as many other parts of the child. As I said before, the cord was quite short, scarcely long enough to reach external to the vulva. But on coming to examine the cord and afterbirth, I found it quite singularly constructed. The cord from the well-formed child was one of the slender, short cords that we occasionally meet with, and joining as it were with the cord coming from the unnatural child, and terminating with but one placenta.

The cord from the unnatural child having quite a singular appearance: not being white and clear looking, as we generally find them, but rather a dark, grumous and apparantly of a gelatinous character; where it emerges from the child is rather higher up than it would be in ordinary children.

We now come to the nates, they having quite the appearance of an ordinary child. The anus appears to be natural; the generative organs are quite developed; and, as Artemus Ward says, we find it belonging to the female persuasion, with vagina and vulva, in fact, possessing all the paraphernalia that is generally accompanying that order.

The length of body, from nates to apex, was $5\frac{1}{2}$ inches; the circumference of the body 13 inches; the thigh being 9 inches in circumference, and $3\frac{1}{2}$ inches in length, containing one bone as it should.

At the joints it had the appearance as if there had been a string drawn tightly around, forming a crevice or stricture as it were.

The length of lower limb, from knee to heel, was 6 inches, and the circumference of the same 5 inches, and containing two bones as usual. The feet and toes were all natural and well-formed. But the child had quite a peculiar color, almost that of a red; but why it should be so I am somewhat at a loss to tell or decide, unless it was caused by the mother attending a ball. After dancing all night, she was taken unwell, and had quite a flow, so much so as to think she was going to miscarry; and perhaps the husband thinking it a time of frolic and revelry, he insisted on his rights as a husband, and she being of a kind turn and accommodating, yielded to his requests. The consequence was conception and cessation of the flow, and nature spliced on and remained till the time of gestation. Perhaps I should have said that the couple had only been married four weeks previous to the time of attending the dance. Hence the flow causing the peculiar color to the deformed child.

